# The social stigma and psychological impact in post COVID 19

**DOI:** 10.1192/j.eurpsy.2023.1661

**Published:** 2023-07-19

**Authors:** N. Halouani, D. Gdoura, O. Bouattour, M. Turki, N. Moussa, S. Ellouze, J. Aloulou

**Affiliations:** 1 Psychiatry “B” Departement; 2 Pulmonology Departement, Hedi Chaker Hospital, Sfax, Tunisia

## Abstract

**Introduction:**

Coronavirus 2019 (COVID19) is a contagious disease. Infected patients are not only the vectors of the disease but also often the victim of the social stigma attached to it.

**Objectives:**

To assess the social stigma perceived by post-COVID19 patients.

**Methods:**

This is a descriptive and analytical cross-sectional study that took place during the period from 1st March to 15th May 2021 with 154 patients who were hospitalized at the COVID19 unit at Hedi Chaker Hospital in Sfax.

The anxiodepressive disorders were screened using the “Hospital Anxiety and Depression Scale”. Post-traumatic stress disorder was assessed using the Impact of Event Scale-Revised.

Perceived stigma due to COVID19 was assessed by items from the psychometric tool: self-reported instrument measuring COVID19-related stigma.

**Results:**

The mean age was 66.62 ± 13.34 years. Male patients represented 60.4% of the study population.

In our study, the prevalence of anxiety, depression and post-traumatic stress disorder was 24.7%, 11% and 13.6% respectively.

In our study, 21.4% of the participants felt discrimination and social stigma, especially from neighbors (18.2%).

Anxiety was statistically correlated with the presence of stigma related to COVID19.

We have a highly significant relationship between depression and stigma. (p=0.002)

No correlation was found between stigma and post-traumatic stress disorder.

**Conclusions:**

In addition to social stigma, people with COVID 19 may perceive themselves as different from normal in terms of physical or mental abilities, leading to a high likelihood of self-stigma and social isolation.

**Disclosure of Interest:**

None Declared

